# Attitudes towards Risk Prediction in a Help Seeking Population of Early Detection Centers for Mental Disorders—A Qualitative Approach

**DOI:** 10.3390/ijerph18031036

**Published:** 2021-01-25

**Authors:** Pauline Katharina Mantell, Annika Baumeister, Stephan Ruhrmann, Anna Janhsen, Christiane Woopen

**Affiliations:** 1 Research Unit Ethics, Institute for the History of Medicine and Medical Ethics, Faculty of Medicine, University of Cologne and University Hospital of Cologne, 50924 Cologne, Germany; annika.baumeister@uk-koeln.de (A.B.); christiane.woopen@uni-koeln.de (C.W.); 2 Cologne Center for Ethics, Rights, Economics, and Social Sciences of Health (CERES), University of Cologne and University Hospital of Cologne, 50923 Cologne, Germany; 3 Department of Psychiatry and Psychotherapy, Faculty of Medicine and University Hospital, University of Cologne, 50931 Cologne, Germany; stephan.ruhrmann@uk-koeln.de; 4 a.r.t.e.s. Graduate School for the Humanities, University of Cologne, 50931 Cologne, Germany; anna.janhsen@caritasnet.de

**Keywords:** health literacy, risk perception, prevention, personalized medicine, help seekers, Big Data

## Abstract

Big Data approaches raise hope for a paradigm shift towards illness prevention, while others are concerned about discrimination resulting from these approaches. This will become particularly important for people with mental disorders, as research on medical risk profiles and early detection progresses rapidly. This study aimed to explore views and attitudes towards risk prediction in people who, for the first time, sought help at one of three early detection centers for mental disorders in Germany (Cologne, Munich, Dresden). A total of 269 help-seekers answered an open-ended question on the potential use of risk prediction. Attitudes towards risk prediction and motives for its approval or rejection were categorized inductively and analyzed using qualitative content analysis. The anticipated impact on self-determination was a driving decision component, regardless of whether a person would decide for or against risk prediction. Results revealed diverse, sometimes contrasting, motives for both approval and rejection (e.g., the desire to control of one’s life as a reason for and against risk prediction). Knowledge about a higher risk as a potential psychological burden was one of the major reasons against risk prediction. The decision to make use of risk prediction is expected to have far-reaching effects on the quality of life and self-perception of potential users. Healthcare providers should empower those seeking help by carefully considering individual expectations and perceptions of risk prediction.

## 1. Introduction

The technical and scientific progress in the field of personalized medicine is paving the way for new insights into the development, treatment and prevention of diseases. Particularly in the field of psychiatry, recent advances in risk prediction are associated with high hopes that a paradigm shift towards disease prevention will be promoted and that a significant reduction in the burden of disease can be achieved in the long term [1,2,3]. Considerable progress has been made, for example, in psychosis research [4,5,6]. Early detection and prevention centers for mental illness have been established in recent decades, following the modern program of predictive, preventive and personalized medicine [7]. 

Key goals of prediction in the context of mental disorders are to identify valid indicators for an increased risk, to predict the onset of illness and to offer risk-adapted prevention measures in order to improve overall health outcomes. This could pave new pathways in disease prevention and thereby lower the burden of mental disorders for those at risk for developing a mental disorder [8,9]. However, prevention methods and goals in public health are critically discussed—especially when it comes to mental health [10]. Mental disorders require specific approaches in distinction to physical illnesses, which must also be reflected in the implementation and design of prevention measures [11]. Furthermore, the handling of Big Data is controversially discussed with regard to sensitive patient and health data [12] and its potential impact on privacy and discrimination risks [13].

The application of such predictive methods based on Big Data approaches in routine clinical psychiatric practice has yet to be tested [14] and requires careful consideration of the expectations and attitudes of those affected. People with mental health problems will be faced not only with the decision of whether or not to make use of risk prediction, but also the need to understand and carefully evaluate the consequences related to the predicted risk information in order to derive self-determined decisions for their health. 

An individual’s health literacy, defined as the knowledge, motivation and competencies to access, understand, appraise and apply health information [15], is widely regarded as one of the key factors for conscious and self-determined health decisions with regard to disease management, prevention and health promotion. The concept of health literacy serves as a theoretical framework for dealing with health-relevant information and making decisions concerning one’s own health. Promoting or improving health literacy is not only about conveying information and developing skills to process health information in order to apply medical measures. It is also argued that improved health literacy is crucial for empowerment [16].

Thus far, the perception and expectations of the general population have been the main focus in research of risk prediction for mental illness, and only a few empirical studies have been conducted on attitudes towards risk prediction of potential user groups in the field of mental health [17]. Little is known about how people deal with probability-based risk knowledge, what expectations they have with regard to risk prediction and what consequences individuals draw in terms of the use of preventive measures. 

The present study aimed to explore the views and attitudes towards risk prediction in a population of people who, for the first time, sought help at early detection centers for mental disorders in Germany. We thereby focused on their individual motives for a hypothetical approval or a rejection of risk prediction to shed light on the perspective of those who are and will potentially be confronted with the decision about using risk prediction. 

## 2. Materials and Methods 

This study was part of a larger survey on health literacy in cooperation with three German university hospitals [18]. The initial survey addressed people who, for the first time, sought help in an early detection center for mental disorders in Cologne, Dresden or Munich between September 2014 and September 2017. Inclusion criteria were initial contact, a minimum age of 15 years, sufficient knowledge of German to complete the question independently and the ability of the interviewees to give their informed consent. Informed consent was obtained from all subjects involved in the study. The research project was ethically approved by the institutional review board of the participating health care institutions (Ethics Committee Medical Faculty, University of Cologne: 14-165; Ethics Committee Medical Faculty, LMU Munich: 93-15; Ethics Committee at the Technical University Dresden: EK 141042016).

Of 310 participants who took part in the health literacy study, *N* = 269 persons responded to the subsequent question about potential use of risk prediction. Sociodemographic and clinical characteristics as well as general level of health literacy are listed in Table 1.

The study included a paper–pencil questionnaire on self-perceived health literacy and two open questions on the views and attitudes towards risk assessment as well as underlying motives for the potential approval or rejection of risk prediction. Health literacy was assessed by the European Health Literacy Questionnaire (HLS-EU-Q47). The HLS-EU-Q is a validated assessment tool including 47 question on accessing, understanding, appraising and applying health information [15]. Methods and results of the health literacy assessment are described in detail elsewhere [18]. 

In deliberate distinction to this quantitative research approach, we used a qualitative design to explore the broad range of subjective risk perception and the views, attitudes and underlying motives for the potential use, i.e., the approval of future risk prediction measures or its rejection without anticipating answers and/or drawing attention to (initially) unconscious aspects [20]. 

The approach of asking about general risk prediction was deliberately chosen to inquire about attitudes towards the researched possibilities of risk prediction in general, including measures to predict physical as well as mental illness. The qualitative data were collected by means of additional open response fields that were embedded in the parent survey, referring to the following question on risk prediction: *“Your personal appraisal: Imagine it would be possible to predict which diseases you will get in your life and how existing diseases will develop. This would require a lot of information, such as your diet, exercise, lifestyle, environmental influences, genetic information (DNA) and images of your organs. If you could use this method to predict the likelihood of contracting a particular disease, would you do so? Please give reasons for your answer”.*

**Figure 1:**
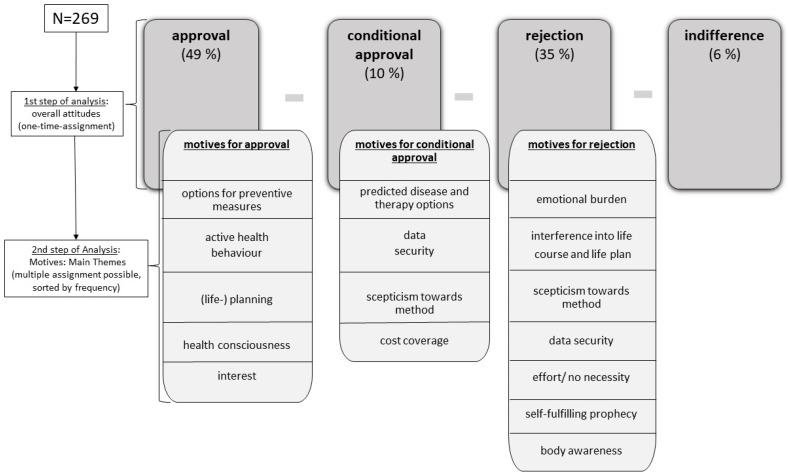
Overview of the two-stage analysis procedure resulting in overall attitudes (approval, conditional approval, rejection, indifference) and the inherent motive categories.

The analysis was carried out in a two-stage procedure using qualitative content analysis (see Figure 1). At the core of the qualitative content analysis stands the step-by-step inductive development of categories alongside the data [21,22]. To ensure content validity, two researchers (PM and AB) independently screened the whole data set and inductively developed a category system. The first set of categories were discussed between the authors and refined throughout the research process, resulting in one common category system. Disagreements were resolved by discussion.

In the first step of the analysis, four overarching themes emerged with respect to the potential use of measures of risk prediction: **approval**—**conditional approval**—**rejection**—**indifference**. In this analysis step, the respective statements were assigned to only one of the above categories, which allowed a percentage distribution of all respondents.

In the second step, we sub-coded the participants’ motives in each of these four categories to understand the reasons for the potential approval, conditional approval, rejection or indifference on whether to make use of prediction or not. Due to the thematic density of the statements, multiple assignment to the corresponding motive categories was possible in this step of analysis. 

Quality criteria of the analysis were achieved in terms of exhaustion and saturation [23,24]. The analysis was carried out using MAXQDA 2018 software [25,26]. 

## 3. Results

### 3.1. Overall Attitudes towards Risk Prediction

Overall, half of the respondents (49%) were in favor of using risk prediction, while 35% would reject such an assessment. In total, 10% indicated that they would make use of risk prediction under certain conditions. Only 6% of the respondents stated that they had no conclusive opinion on the potential use of risk prediction and expressed themselves indifferently (see Figure 1).

Most participants took a very clear and unequivocal position on whether they would approve or reject risk prediction. The different perspectives that underlie this overall attitude were reflected in the respective answers. Proponents who were in favor of using risk prediction, for example, replied: *“Of course, it is very important to get knowledge about your current and future state of health”*, *“Yes, it would be stupid not to do it”* or *“100%, because I would rather disclose private data than risk a possible illness”*. Equally strong were the responses related to a rejection of predictive risk assessment. For example, the answer of one interviewee was *“No, because knowing about the risk would drive me crazy”*. Another participant replied: *“No, because then I’ll be just waiting to become ill”*.

The participants perceived the anticipated benefit of a risk prediction differently. The handling of risk knowledge as well as the consequences of action resulting from this knowledge seemed to be very individual. The question of the potential use showed a strong positioning of the participants, which was partly based on normative arguments, revealing different beliefs and health or life expectations of the interviewees. That highlights the different views and attitudes on risk prediction, which affect the anticipated appraisal of the risk information resulting from it and, in turn, the decision on its potential use.

### 3.2. Motives

The following section shows the central motives that were reflected in the decisions to approve, conditionally approve or reject the potential use of predictive measures. The respective motive category is printed in bold; a full overview of all categories is shown in Figure 1.

#### 3.2.1. Motives for an Approval of Predictive Measures

The most frequent reason given by participants in favor of risk prediction related to the possibility to make use of preventive measures in order to reduce risk and thereby prevent the onset of a disease. This category was labelled **option for preventive measures**. Participants in this category largely formulated their responses impersonally in the sense of “one can” instead of “I can”, e.g., *“Yes, because in case of a positive result you can initiate preventive measures at an early stage”*. It often remained unclear whether the person concerned played an active or passive role in the implementation of preventive measures and behavior. The ambition to make use of risk prediction seemed to lie in the subsequent option to apply the information by making use of preventive measures. The statements indicate that it remains to be seen whether the predicted risk would inevitably result in the actual use of these measures. Following the statements of the interviewees, participants would (re)consider the use of preventive measures on the basis of the predicted risk and the available methods for illness prevention.

In contrast to this, the category **active health behavior** summarized statements of persons who indicated that they would make use of risk prediction in order to use the risk knowledge to actively engage in their own health, e.g., by changing their lifestyle to reduce the risk of developing the disease. One participant answered, *“Since I am a person who is very health conscious, I would use this procedure. That way I could adapt my lifestyle to it”*. Proactively drawing consequences of action from the risk probability is already part of the answers in this motive category.

Other interviewees indicated that they are in favor of using risk prediction because it allows them to adapt their **(life) planning**. In this context, some of them explicitly expressed the desire to maintain control over their lives. Examples of statements assigned to this category were the following: *“I would use this procedure because it allows you to adjust your life plan accordingly”* and *“[…] so I can prepare myself for any illness that may arise”*.

Thus, the desire for control and self-control of one´s own actions turned out to be a central common feature of the statements on **active health behavior** and **(life) planning**.

Answers assigned to the category **health consciousness** were characterized by the necessity of being informed about one’s own health status. One participant stated that *“it is of course very important to get knowledge about your state of health”*. Some statements in this category reflected the inherent hope that knowing a certain risk would contribute to an increased (self-)awareness of one’s own body and better health consciousness in general. 

A further motive for making use of prediction in the future was the participants’ **interest** in such measures as well as a certain curiosity about the method of risk assessment and the new pathways resulting from it in general. 

#### 3.2.2. Motives for a Conditional Approval of Predictive Measures

The majority of respondents who indicated that they would make use of risk prediction solely under certain conditions stated that their decision depended on the **predicted diseases and therapy options** available. Participants answered, for instance, *“For diseases with treatment options that would limit the quality of life severely without treatment, I would accept the offer. However, if the quality of life is only slightly limited, or if the disease is untreatable, the information would probably be a much greater burden […]”*.

Interestingly, further categories related to the main category **conditional approval** included motives which were partly also mentioned as reasons for the rejection of prediction but were, in contrast, formulated as conditions in this context. These included **data security** *(“I would do this only if the data is collected with my consent and remains absolutely private.”)*, **skepticism towards the method** (*“I would use it only if this approach has reached the necessary maturity.”)* and the certainty of **cost coverage** of the predictive measure by their health insurance *(“Yes, if it’s free of charge.”)*. 

#### 3.2.3. Motives for a Rejection of Predictive Measures

A majority of those respondents who were opposed to risk prediction stated that they were concerned that knowing about a certain risk could have negative effects on their mental well-being. Statements referring to mental stress based on the information of a predicted risk were summarized in the category **emotional burden**. Statements by respondents assigned to this motive category included the following: *“No, I would rather not use it. I always worry too much about my body and a prediction of a possible disease would be an extreme psychological stressor for me. For me, personally, the disadvantages outweigh here.”*; *“No, the probabilities would scare me.”*; *“No, it would unsettle me too much and make me panic constantly”*. Within this category, respondents frequently gave subjective answers including narratives and biographical references. Some interviewees explicitly referred to their mental state, e.g., they spoke of an already *“strained mental state”* or an *“unstable psyche”*, which would be further strained by an outcome of a higher risk. Feelings of stress and fear of the future were other personal reasons given in this context. In contrast to other categories, e.g., **options for preventive measures** or **predicted disease and therapy options**, statements within this category included personal reference and the individual life situation and were cited as the main motives for rejection of risk prediction.

Furthermore, the knowledge about a risk was perceived as a possible **interference in life course and life planning**. The interviewees indicated that they fear the knowledge about an increased risk, because it would influence their life planning in a negative way. One participant stated, for instance, *“No, predictions could influence my life plan to such a degree that it would proceed differently, if this prediction had not taken place.”*, while others replied, *“No, I wouldn’t use that, because I’m convinced that life shouldn’t be planned”*.

The category **self-fulfilling prophecy** included answers in which respondents expressed a suspicion or concern that knowledge of a risk might lead to its fulfilment and thus increase the actual risk. Furthermore, respondents feared that knowledge of a risk alone could make them ill: *“I wouldn’t do it because I think that fear of a possible or probable disease could make you sick, even if you don’t end up with the disease in question”*.

Overall, both categories, **interference in life course** and **life planning** as well as **self-fulfilling prophecy**, represent a clear contrast to the categories **active health behavior** and **(life) planning**, which were presented as motives for approval of risk prediction. Here, the fear of losing control seems to be an essential decision component for rejection.

Another motive for rejection was based on mistrust in the concept and methodology of risk assessment. The motive category included statements related to **skepticism towards the method** of risk prediction. Respondents indicated that they did not believe that reliable statements on risk could be made with the aid of the latest technologies. 

Some interviewees substantiated their rejection with concerns about **data security**. In this respect, the respondents mentioned on the one hand, a lack of confidence in the technology/IT, with which the patient data would be aggregated and analyzed (e.g., *“No, I’m very skeptical about personal data retention and the technology behind it.”)*. On the other hand, they claim a lack of confidence in the people (doctors, researchers, etc.), who would ultimately handle the data (e.g., *“No, I’m a dedicated data protector and do not want to entrust this data to anyone. The best data are no data collected at all. […] The industry standards of privacy are ridiculous.”)*.

Other respondents rejected risk prediction because they did not wish to undertake the **effort** that a prediction or a potentially increased risk would require and/or simply did not see the need for such a measure. A further motive, which made the interviewees reject risk prediction measures, was the prioritization of their own **body awareness**, which, according to their own statements, they preferred to rely on, rather than on the analysis of data.

## 4. Discussion

Acceptance of predictive measures is an essential requirement for the successful implementation of measures to prevent mental illness. This study shows the variety of attitudes towards risk prediction in a population of people with perceived mental health problems. The consideration of hypothetical individual perspectives and motives in favor of or against risk prediction are particularly important in this population, since recent research demonstrates the feasibility of developing risk prediction models for psychiatric disorders and clinical practice is moving towards embracing prediction and prevention [8]. Thus, people with mental health problems may be increasingly confronted with the decision to choose for or against risk prediction in the near future. 

The statements of the interviewees show that the expected benefit of risk prediction is perceived very differently by individuals. Reasons and motives behind the attitude for potential use (approval or rejection) illustrate the diverse views and expectations towards risk prediction. 

Above all, an **emotional burden** was stated as a central reason against the use of risk prediction, which was thought to be caused by the risk knowledge rather than the potential illness itself. With regard to the study population, we expected that people with current mental health problems may have a particularly high level of attention in terms of possible negative emotional implications. For future research it would be of interest to know whether those who use risk prediction also include the potential harm of emotional burden in their personal risk–benefit analysis. With regard to the population of help seekers, it must be considered that these people are generally not healthy or free of complaints. In moments of help seeking, most already have first symptoms that can show the onset of a mental illness. They may turn to an institution that provides diagnostic and risk clarification. It is therefore all the more surprising that some respondents were vehemently opposed to the risk prediction. At the same time, it should be borne in mind that pre-existing symptomatology, such as depressive symptoms, which affected more than 70 percent of this sample (see Table 1), can have a negative impact on the current future prospects and plans of those affected. However, the justifications were, for the most part, written in a differentiated manner and their content partly indicated a personal risk–benefit analysis that forms the basis of the decision-making process for or against the use of risk prediction. The given motives for the potential use of risk prediction point to far-reaching consequences for users—both for one’s own self-image and for shaping one’s own life. 

Overall, the differentiated responses reflected different attitudes toward risk prediction, which did not seem to be positively or negatively influenced by the presence of current mental health problems per se. In this respect, it should be noted that participants were, on average, of a relatively young age and had a high level of education (see Table 1).

Furthermore, it became clear that the desire for self-determination and personal responsibility is of great importance, both for the motives of rejection and for the approval of risk prediction. This is reflected, for example, in the motive of **(life) planning**, which was emphasized in favor of risk prediction. Statements by these respondents indicated that they would make use of the predicted risk and all the associated trade-offs in order to plan for their future in a self-determined manner. In contrast to this, the desire for self-determination became equally clear in the category **intervention in the life course and life plan**, which was a motive for rejecting risk prediction. Both positions convey different values with regard to personal life plans, beliefs and health expectations. Another key example that illustrates these fundamentally different beliefs and approaches to risk prediction is the desire for control of one’s own life. On the one hand, there seems to be a wish for risk knowledge in order to specifically counteract an existing risk through **active health behavior** and **(life) planning**. On the other hand, the fear of loss of control was mentioned as a motive against the use of risk prediction as it was formulated, for example, in the motive of **self-fulfilling prophecy**. Fears of loss of control, as reflected in several categories, could be indicative of internal processes on the part of respondents who reflect concerns of social stigma when deciding for or against risk prediction. In the clinical setting, these concerns may be influenced by the social desirability to risk prediction, namely unexpressed (hidden or even unconscious) motives on the part of clinicians or researchers in favor of risk prediction. From a clinical point of view, giving consent to risk prediction may seem desirable to prevent the undesirable occurrence of the mental disorders. These pre-assumptions could create subtle pressure and force the fear of stigmatization for people concerned in the decision-making situation, which eventually is a major ethical problem.

The results show that the respondents who sought help at an early detection center for mental disorders were driven by very different motives when dealing with risk knowledge. The decision for approval or rejection of risk prediction seems to be strongly dependent on individual values and concepts. Thus, the evaluation basis of a risk prediction seems to go far beyond an understanding of objective medical facts. From an ethical perspective, health care providers should take these individual values into account and support a personal risk–benefit analysis when it comes to decision-making about the use of risk prediction in the context of mental health and beyond. 

Concurrently, this process may be particularly challenging for people with mental health problems. The parent study on self-assessed health literacy has shown that those affected find it particularly difficult to critically appraise health-relevant information [18].

In line with these findings, the latest evidence on health literacy showed that people with mental illness experience particular challenges in dealing with health information [27,28,29].

Following up on the statistical analysis of the parent study on health literacy among people with mental health problems [14], the intention to make use of future risk prediction or not was not significantly associated with the health literacy levels of the respondents This finding indicates that the decision whether to make use of such future measures is highly individual and relies on personal values, preferences and feelings regarding Big Data approaches on the one hand and the perceived benefit of risk prediction on the other hand. These very personal reasons should be considered separately from individual skills and abilities to process health information.

Studies from other medical fields such as breast cancer [30], heart failure [31] and diabetes [32] also impressively showed that risk prediction and risk knowledge are perceived very differently and can have manifold consequences for the individual. Blakeslee et al. (2017) stress that “accepting patients’ experiences and beliefs in their own right and letting them guide the discussion may be important for a satisfying decision-making process” [30] (p. 2353). In contrast to somatic diseases, the risk prediction of mental illnesses is accompanied by disease-specific challenges. While somatic diseases are communicated relatively openly in society, mental illnesses have long been tabooed. While there has been increasing public attention to mental health in recent years, people with mental illness continue to face stigmatization and exclusion, which can make their participation in society considerably more difficult or even prevent it altogether [33]. 

From an ethical perspective, the question arises how risk knowledge can influence affected persons and their environment. The interviewees anticipated far-reaching consequences of risk prediction for their personal well-being and their life paths. Negative social consequences that could result from this risk knowledge, such as insecurities and prejudices in the social environment or possible consequences for family members that would result from a genetically increased risk, were not explicitly addressed in this population. According to labelling theories of psychiatric stigmatization, a positive test result on an increased risk could lead to early stigmatization of those affected [34]. Persons who are at an increased risk of mental illness could thereby experience similar stigmatization and discrimination as those who are already ill. However, not only stigmatization of third parties but also self-stigmatization is significant in this context. Initial study results show that knowledge of an increased risk can also trigger increased stigmatization stress and self-labelling as “mentally ill” in those affected, which leads to negative effects on their well-being [35,36]. At the same time, clinical practice has shown that risk labelling can also lead to psychologic relief of the affected persons, since the previously irritating, uncontrollable and inexplicable complaints can then be assigned to a disease model [33].

The protection of privacy and the sensitive handling of personal health information are rightly emphasized in this context; published risk information (e.g., an increased risk of schizophrenia) can negatively impact individual circumstances, such as insurance relationships or decisions in hiring an individual at increased risk [37]. The challenge is to find a viable way to protect individuals’ health data while enabling the flow of information necessary to promote quality health care [38].

As the results of this study show, dealing with and deciding upon risk prediction cannot be broken down to clinical parameters from an objective perspective. On the contrary, individual expectations and perceptions of risk prediction must be considered to maintain or improve the quality of life of an individual. 

Health literacy in people with mental health problems is critical, especially with regard to appraising health information [18]. Meanwhile, the individual preference as a result of weighing up the advantages and disadvantages of risk prediction is becoming increasingly important, as knowledge of a risk can already have far-reaching consequences for an individual’s lifestyle and quality of life. Interventions to support the decision-making process and dealing with risk knowledge should focus even more on the promotion of competencies to appraise health information against the background of personal values and life concepts. On the basis of the available results, quantitative assessment approaches should be developed to deepen knowledge on subjective risk perception and risk expectations in the context of mental health to facilitate decisions on risk prediction in clinical practice.

Decisions on risk prediction require all the more successful communication between the healthcare professional and the person at risk, including comprehensive information on the procedure of risk prediction and its potential consequences for the individual. This is of particular importance for people with low health literacy, who need to be appropriately supported by healthcare professionals in the process of decision-making on the basis of all relevant information. In light of other findings on the lack of understanding probability values even among clinicians [39], training and education of medical personnel should be considered to ensure adequate communication of options and results of risk prediction. The increasing possibilities of risk prediction and the resulting options for action raise hope for the development of sustainable prevention opportunities for people with mental illness. 

Dealing with risk knowledge, however, must be implemented sustainably by decision-makers, thereby considering individual risk attitudes in order to ensure a high individual benefit. These results can be seen as a call for action for health care policy makers to include, beyond clinical parameters, the personal resources and values of patients for or against the use of risk prediction in medical decisions. 

Study participants were recruited from three early detection centers, where young people and people with a high level of education were significantly over-represented compared to other populations with mental disorders. Results in the investigated group should, therefore, be regarded as explorative and a bias of the selection of “help seekers” should be considered when interpreting the results. It is of note that of the 310 participants who took part in the parent study on health literacy, 41 did not respond to the question on risk prediction. Possible reasons could be that the question was asked at the end of the survey and, unlike the previous questions, was open-ended and required written responses. In addition, the participants surveyed may have found the question complex and difficult to answer. This may also indicate that the risk prediction decision is not trivial and may overwhelm some potential users. 

Limitations of this study are that a *hypothetical* use of risk prediction *in general* but not specifically related to mental disorders was queried. We surveyed a population at increased risk (in this case, people with increased risk for mental disorders) potentially facing risk prediction options within their initial contact with an early detection center. However, asking about general risk prediction might have influenced the results in two ways. Firstly, studies show that the intention of health-related behavior and theoretical preference may differ from actual behavior. Nevertheless, regardless of actual utilization, there is clear evidence about how different individual preferences and anticipated consequences are perceived with respect to risk prediction. Secondly, it is not clear whether the participants related their answers to certain physical or mental illnesses, which might have influenced their answers. Against the background of societal discrimination and stigmatization tendencies towards mental disorders, it cannot be excluded that a risk prediction focused on mental illnesses would have been met with even greater rejection among the respondents. However, the main purpose of this study was to investigate the manifold views and attitudes towards risk prediction approaches in general. Attitudes towards predicting physical illness are of no less interest in this population, as physical comorbidities are very common in people with mental illness. This study aimed to draw an initial picture of the views and attitudes of potential users with mental health problems. Future studies should also include other consumer groups to get a deeper understanding of the needs and preferences of those concerned. 

Qualitative content analysis is based on interpretative analyses that do not aim to draw quantifiable conclusions on statistical relationships. However, qualitative research enables access to reality through subjective opinions, perspectives and underlying interpretative processes [20]. In the context of risk prediction, the subjective meaning is given a particularly great significance to determine naturalistically what attitudes participants have towards risk prediction. The results can be seen as an important starting point for further investigations of affected persons’ perspectives, which can contribute to a differentiated implementation of risk prediction in clinical practice.

## 5. Conclusions

The new possibilities of Big Data-based risk prediction promise a rapid upswing in research of new predictive, preventive, diagnostic and therapeutic approaches to care. In the field of mental health, this approach is also associated with high hopes for a paradigm shift from treatment of illness to illness-prevention and health-promotion. Thus far, little research has been conducted on potentially affected individuals’ values and preferences regarding risk prediction.

This study showed that persons with mental health problems tend to have clear preferences for the use or rejection of risk prediction. Preferences were accompanied with various and sometimes contrary motives. The anticipated impact on self-determination was, in particular, a driving decision component, regardless of whether the person in question would decide for or against risk-profiling. 

At the same time, the results of this study show that decisions on risk prediction are expected to have far-reaching effects on the quality of life and self-perception of potential users. A differentiated view of possible advantages and disadvantages of risk prediction, considering individual expectations and values, seems indispensable. This is especially true in the context of mental disorders, where public and self-stigma is still alarmingly present. The results of such risk prediction approaches could reinforce such tendencies including discrimination. These implications should be taken into account when considering the development and implementation of risk prediction in the psychiatric context.

## Figures and Tables

**Table 1:** Characteristics of study participants.

**Study Participants (*N* = 269)**	
**Age**, years, mean (SD)	24.75 (5.3)
(min–max)	(15 to 41 years)
≤18 years, no. (%)	21 (7.8)
19 to 25 years, no. (%)	139 (51.7)
26 to 30 years, no. (%)	69 (25.7)
31 to 41 years, no. (%)	40 (14.9)
	*n* = 269
**Sex**	
Female, no. (%)	105 (39.0)
Male, no. (%) No information	163 (60.6) 1 (0.4)
	*n* = 269
**Educational degree**	
No degree/lower secondary education (“Hauptschulabschluss”), no. (%)	25 (9.3)
Medium secondary education (“Mittlere Reife”), no. (%)	58 (21.6)
Upper secondary education (“(Fach-) Hochschulreife”), no. (%)	133 (49.4)
≥ University degree, no. (%) Unknown	52 (19.3) 1 (0.4)
	*n* = 269
**Migration background**	
Yes, no. (%) No, no. (%) Not specified by participant	126 (46.8) 105 (39.0) 38 (14.2)
	*n* = 269
**Psychopathology** (according to ICD-10) ^1^	
Depressive disorders, no. (%)	104 (38.6)
Schizophrenia, schizotypal and delusional disorders, no. (%)	48 (17.8)
Neurotic, stress-related and somatoform disorders, no. (%)	37 (13.8)
Others No diagnosis unclear	45 (16.7) 6 (2.2) 29 (10.8)
	*n* = 269
**Increased risk for psychosis** (yes), no (%)	56 (20.8) *n* = 269
**Level of depression** (according to BDI-II) ^2^	
No depression (or remitted) (scores 0–13), no. (%)	72 (29.3)
Mild depression (scores 14–19), no. (%)	38 (15.5)
Moderate depression (scores 20–28), no. (%)	77 (31.3)
Severe depression (scores 29–63), no. (%)	59 (24.0)
	*n* = 246
**Level of Health Literacy** (according to HLS-EU-Q47) ^3^	
General HL, M(SD)	31.25 (07.15)
	*n* = 267

^1^ ICD-10: International Statistical Classification of Diseases and Related Health Problems. ^2^ BDI-II: Beck Depression Inventory II [19]. ^3^ European Health Literacy Questionnaire [15]: Thresholds were set to compare levels of HL, divided in excellent (>42–50), sufficient (>33–42), problematic (>25–33) and inadequate (0–25). The bold words show the category of the following subcategories in this column.

## Data Availability

The data presented in this study are available on reasonable request from the corresponding author.
